# Balance Testing in Multiple Sclerosis—Improving Neurological Assessment With Static Posturography?

**DOI:** 10.3389/fneur.2020.00135

**Published:** 2020-02-26

**Authors:** Hernan Inojosa, Dirk Schriefer, Antonia Klöditz, Katrin Trentzsch, Tjalf Ziemssen

**Affiliations:** MS Center, Center of Clinical Neuroscience, Department of Neurology, Carl Gustav Carus University Hospital, University of Dresden, Dresden, Germany

**Keywords:** balance, multiple sclerosis, static posturography, expanded disability status scale, sensitivity, Romberg test

## Abstract

**Background:** Balance problems can severely limit the quality of life for people with Multiple Sclerosis (pwMS) already in the early stages of the disease. PwMS are usually assessed with the Expanded Disability Status Scale (EDSS), which includes a Romberg test for assessing balance. As the EDSS assessments are subjective to the examining neurologist, the postural stability of pwMS could be objectively quantified by implementing static posturography to detect balance problems and address preventive medical care.

**Methods:** In this cross-sectional study, we added static posturography to the neurological EDSS examination in pwMS and healthy subjects to determine how this technique could supply additional information during the evaluation of the cerebellar functional system of the neurostatus EDSS as clinical outcome already in early disease stages. Static posturography was performed with subjects standing on a force platform while outcome variables such as delineated area, average speed and average sway were obtained. Unpaired *t*-test as well as (Welch's) analysis of variance (ANOVA) with pairwise *post-hoc* comparisons according to Games-Howell were used. Spearman rank correlations were implemented to study associations of balance outcomes with EDSS-associated outcomes.

**Results:** A total of 99 pwMS (mean age: 35.01 years; EDSS median: 2.0, 68.69% females) and 30 healthy subjects (mean age: 34.03 years; 70% females) were enrolled. PwMS had worse performances in the three evaluated balance parameters than the healthy group (all *p* < 0.001). Even patients without postural instability as documented in the Romberg test score of the EDSS assessment showed significantly worse outcome regarding the delineated area [+1.97 cm^2^, 95%-CI (0.61–3.34); *p* = 0.002] vs. healthy controls. Similar results were observed for the comparison between pwMS with normal cerebellar function EDSS-systems and healthy subjects. There were significant correlations with the EDSS, cerebellar function score and Romberg test for the delineated area and average speed (*r*'s ranging from 0.330 to 0.537, *p* < 0.001).

**Conclusions:** Static posturography can complement neurological assessment of EDSS as an objective and quantitative test, especially for MS patients in early stages of the disease.

## Introduction

Multiple sclerosis (MS) is a chronic inflammatory immune disease well-known due to the heterogeneity of its clinical manifestations (1). Among them, deficits in balance are often present even in early stages of MS. In later disease stages, they are the primary cause of falling associated with further injuries (2–5). Balance itself is defined as the ability of maintaining the body center of gravity with minimal sway (6). Up to two thirds of MS patients report incapacitating balance or coordination problems in their daily life (7).

While it was initially assumed that lesions in the cerebellum were the main cause of gait and postural instability (8), it is now considered that a slowed transmission of somatic sensory impulses may have an important effect on the postural stability of MS patients (7, 9–11). A combination of central and peripheral components with afferent and efferent signals modulates the balance (7, 12). Limitation of the sensitive receptor function of muscle spindles, Golgi organs or joints is as important as the impulse transmission via peripheral nerves to the spinal cord (13, 14).

Fear of falling and its consequences dramatically limit MS patients' quality of life and can often lead to reduced activity levels, decreased productivity and social withdrawal (5, 7, 15). The inability to appropriately organize sensory information can lead to an exacerbation of impairments and to a certain selection of movement strategies to compensate for these deficits (4, 15). It is therefore of special interest to promptly identify balance dysfunction in MS patients, so appropriate medication, physiotherapy or rehabilitation strategies can be formally prescribed to minimize disability (7, 16, 17).

Currently, the examination of MS patients is supported by different neurological tests and scales (18, 19). The most used disability scale in MS is the Expanded Disability Status Scale (EDSS). This scale is well-established among neurologists, although it has been widely criticized due to its psychometric characteristics, including a poor reliability or responsiveness (18, 19). The subjective assessment of certain functional domains, especially those with barely perceptible clinical signs or low disability, may make the characterization of MS patients difficult. As part of the EDSS, a complete neurological examination is performed. The evaluation of the cerebellar functional system of EDSS includes the Romberg test, which provides orientation on pathology in the proprioceptive pathway, especially in the dorsal columns of the spinal cord (4). It can be carried out with both open and closed eyes. Approximately 10 to 20 s after closing the eyes, there is a physiological increase in swaying. A stable standing position shows that at least two of the three postural control inputs are intact (20). Limited postural control is manifested by an increase in the patient's swaying perceptible to the examiner. If an increase in swaying occurs only during absence of visual stabilization mechanisms, the pathology is suspected to be in the proprioceptive system of the body (20–22). A pathological result is however not specific for multiple sclerosis and occurs in several neurological diseases (e.g., diabetic polyneuropathy, vitamin B_12_-deficit or alcohol-intoxication) (20).

Currently, this postural instability has to be subjectively rated by the neurologist and documented with the cerebellar functional score of the EDSS. Nevertheless, the Romberg test can be quantified and further evaluated using specialized technology to obtain quantitative and objective results. Examples are static and dynamic posturography using force platforms (7).

There have been numerous successful attempts to assess postural instability in MS patients (12, 23, 24), some of them to determine risk of falling (25–27) or to establish correlation with disease disability (26–29). A better sensitivity for static posturography than the classical Romberg test has been previously reported, even with a possible prognostic value (24). However, despite intensive research, a standardized measurement algorithm has not been established yet. Static posturography involves the electronic evaluation of the body's center of pressure or gravity, recording a wide range of more than 100 balance-relevant parameters including speed, sway, root mean square distance, delineated area or 95% confidence ellipse as well as other values (7). Even though the choice of the ideal static posturography outcome measure could be problematic due to the immense amount of available variables (30), static outcomes equivalent to delineated area, average sway and average speed of sway calculated from mediolateral sway amplitude have been shown to be the strongest predictors to discriminate impaired people with MS from healthy subjects according to the results of a machine learning approach (25).

In this study, we aimed to assess how static posturography techniques could quantify balance dysfunction in MS patients and add quantitative objective information to the EDSS. Different parameters of static posturography were compared in MS patients and healthy subjects. In addition, these parameters were put into context to the Romberg test performed as part of the EDSS Neurostatus (31).

We hypothesized that MS patients, even those with low or no clinically detected disability, would have worse performance in balance parameters than healthy subjects. The aim is to add static posturography as an objective functional test to the neurological EDSS assessment in the early stages of MS (EDSS range < 4).

## Methods

We conducted a cross-sectional study in the Multiple Sclerosis Center at the Center of Clinical Neuroscience at the Department of Neurology, University Hospital Carl Gustav Carus, Dresden, Germany. Patients with MS (PwMS) and healthy subjects (HS) without neurological disease were invited to participate. Inclusion criteria were as follows: (1) confirmed diagnosis of Multiple Sclerosis, (2) EDSS Score between 0 and 5.0, (3) age between 18 and 50 years, (4) no acute attacks or cortisone treatment in the preceding 3 months period and (5) written informed consent. Each participant was examined according to good clinical practice (GCP) guidelines. The study was approved by the local ethics committee.

### EDSS Neurostatus

PwMS underwent a full neurological examination by Neurostatus-qualified neurologists from our MS center in Dresden to calculate EDSS scores and to exclude proprioceptive or orthopedic impairment. Only patients with unrestricted ambulation and fully ambulatory patients, according to neurostatus scoring definitions, were enrolled (32).

As part of the examination, all seven functional systems and ambulation were evaluated. An important comparator of this study was the cerebellar functional system with focus on the Romberg test. The rating of the whole cerebellar functional system ranges from 0 (normal examination) to 5 (unable to perform coordinated movements due to ataxia), with 1 step intervals. Similarly, the Romberg Test is scored with 0 (normal), 1 (mild), 2 (moderate), and 3 (severe) as instructed by the neurostatus training material.

For further evaluation, PwMS were classified into three EDSS subgroups to assess the association between the degree of balance dysfunction and disability documented by EDSS. According to neurostatus scoring guidelines, patients with an EDSS step of 0–1.5 have no clinical disability with or without minimal signs of the disease. By EDSS step 2.0–2.5, minimal disability can be observed in up to two functional systems. With EDSS Step scores ≥ 3.0, a higher degree of disability and impact on daily activities are present.

### Static Posturography

The static posturography examination was performed by a computer-driven coordination and balance analysis device that is suited for clinical use (Force Platform GK-1000, MediBalance Pro Test- and Trainingssystem, MediTECH Electronic GmbH). This platform has four piezoelectric sensors installed which measure the position of the patient's center of gravity and its variations converting pressure in electric impulses. A PC with a software package included with the Force Platform was connected to the platform, including a diagnosis software with multiple measurement capabilities. With electronic amplification, coordinates for the center of gravity were automatically calculated on a two-dimensional plane (33).

A trained individual gave proper instructions to the subjects for a standardized assessment and was not privy to the disease diagnosis. To assess the subject's balance, static posturography was performed using the Romberg test's position. Subjects were asked to stay upright and barefoot on a corresponding marked area upon the measurement platform as stable as possible. Patients stood with a standardized position with their feet separated using a track width of 10 cm and with horizontally raised arms in front of them with palms facing up as a provocation and distraction mechanism (20). Each measurement was started after a sufficient adjustment period of 20 s standing on the electronic platform with closed eyes and had a duration of 30 s. Measurements were performed with closed eyes to emulate the balance conditions adopted by the patients during the evaluation of the Romberg Test as part of the cerebellar function system score of the EDSS. Retiring visual stimulation may uncover masked proprioception or sensory disorders that may be present within pwMS (20).

### Balance Outcomes

The balance parameters assessed by static posturography were defined as follows:

- Delineated area: described surface during the measurement of the center of gravity of the subject. Continuous triangles from the mean value of all measurement values of the last point to the current measurement point are calculated (>95% confidence interval). Points on the grid which overlap numerous times are not counted more than once (measured in mm^2^).
- Average sway: average distance of all measurements from the center of all measurements (in mm).
- Average speed: average speed at which the central pressure point of the subjects moves on the platform (measured in mm/s).

These outcome measures were automatically generated by the commercially distributed Force Platform GK-1000 as mediolateral sway measures.

### Statistical Analysis

Normality of data was assessed visually using quantile-quantile plots and confirmed with Shapiro–Wilk tests. Parametric analyses were used, unless otherwise stated. To stabilize variance and to optimize normality for (slightly) right-skewed distributions of balance outcomes, balance variables were log transformed before analyses. Quantitative population characteristics were presented as measures of central tendency (mean, median), followed by standard deviation (SD). Categorical characteristics were expressed as relative frequencies. In the evaluation of balance parameters, a descriptive specification of (crude) mean values and standard deviations occurred. Comparisons between MS patients and healthy subjects were made with unpaired *t*-test and Chi-squared tests, accordingly. To evaluate mean differences between population subgroups (healthy subjects and patient subgroups according to EDSS, Cerebellar FS or Romberg test), variance of analysis (ANOVA) was carried out. In case of not achieving variance equality (as indicated by Levene's test), Welch's ANOVA was used. In case of statistically significant ANOVA results, Games-Howell *post-hoc* test was conducted to compare means of subgroups pairwise, as Games-Howell does not assume equal sample sizes (nor variances). Spearman rank correlations were calculated to study bivariate relations of balance outcomes with EDSS, Cerebellar FS and Romberg Test results. Significant results were those with (adjusted) significance levels of *p* < 0.05. All statistical analyses were performed using IBM SPSS version 25.0 (IBM Corporation, Armonk, NY, USA).

## Results

A total of 129 study participants (mean age 34.78; 69% female; 99 PwMS and 30 corresponding HS) were examined. The mean age of the PwMS group was 35.01 years (SD 8.21), 68.7% were female, with a median EDSS of 2.0 on a range from 1.0 to 5.0. The average time since MS diagnosis was 5.5 years (SD 4.62). No patient presented an EDSS score of 0. The HS group presented a mean age of 34.03 (SD 7.99) years with 70% of female gender. Both groups differed neither in age (*p* = 0.893) nor in gender ratio (*p* = 0.563).

### Cerebellar Function Score in Neurostatus EDSS Examination Including Romberg Test

PwMS had a mean cerebellar function score of 0.74 (SD 0.78; median 0), ranging from 0 to 3; 42.42% of patients had a normal cerebellar function (with a score of 0), 41.4% of 1, 12.1% of 2, and 4% of 3. For the Romberg test, the mean score was 0.32 (SD 0.55; median 1), with scores between 0 and 2 points (71.7% had a score of 0, 24.2% of 1 and 4% of 2).

### Static Posturography in MS Patients and Healthy Subjects

Table 1 shows the static posturography results of MS patients and healthy subjects. Significant differences between both groups could be observed in the three evaluated parameters, namely: delineated area pwMS vs. HS (5.48 cm^2^, SD 7.65 vs. 1.67 cm2, SD 0.98; *p* < 0.001), average sway (16.04 mm, SD 7.59 vs. 12.94 mm, SD 6.75; *p* = 0.047) and average speed (24.40 mm/s, SD 14.66 vs. 16.22 mm/s, SD 3.97; *p* < 0.001).

**Table 1:** Balance parameters in healthy subjects and PwMS.

	**Healthy (*****N*** **=** **30)**		**PwMS (*****N*** **=** **99)**		
**Balance parameters**	**Mean**	**Standard deviation**	**Mean**	**Standard deviation**	***p***
Delineated area (cm^2^)	1.67	0.98	5.48	7.65	<0.001
Average sway (mm)	12.94	6.75	16.04	7.59	0.039
Average speed (mm/s)	16.22	3.97	24.40	14.66	<0.001

### Balance Parameters According to EDSS Step Score

The balance parameters were also analyzed according to EDSS subgroups. Subgroup EDSS 3.0–5.0 differed in all three outcome parameters from the healthy group (Table 2). Further, for delineated area and average speed, the subgroups EDSS 2.0–2.5 and EDSS 0–1.5 also differed from the healthy group, whereas average sway difference from these subgroups did not reach statistical significance.

**Table 2:** Balance parameters in healthy subjects and MS patients according to EDSS Step Score.

**Groups (*N*)**	**Healthy (*****N*** **=** **30)**		**EDSS 0–1.5 (*****N*** **=** **40)**		**EDSS 2.0–2.5 (*****N*** **=** **30)**		**EDSS 3.0–5.0 (*****N*** **=** **29)**		
**Balance parameters**	**Mean**	**Standard deviation**	**Mean**	**Standard deviation**	**Mean**	**Standard deviation**	**Mean**	**Standard deviation**	***p* (ANOVA)**
Delineated Area (cm^2^)	1.67^b^^,^^c^^,^^d^	0.98	3.46^a^^,^^d^	4.07	4.00^a^	2.86	9.79^a^^,^^b^	12.09	<0.001
Average Sway (mm)	12.94^d^	6.75	14.11	7.20	15.64	6.06	19.11^a^	8.72	0.025
Average Speed (mm/s)	16.22^b^^,^^c^^,^^d^	3.97	21.39^a^^,^^d^	10.65	20.96^a^^,^^d^	7.83	32.12^a^^,^^b^^,^^c^	21.06	<0.001

a *significant difference with healthy group (p < 0.05) in post-hoc analysis*.

b *significant difference with EDSS 0−1.5 group (p < 0.05) in post-hoc analysis*.

c *significant difference with EDSS 2−2.5 group (p < 0.05) in post-hoc analysis*.

d *significant difference with EDSS 2.5−3.0 group (p < 0.05) in post-hoc analysis*.

Patients with an EDSSscore between 0 and 1.5 showed significant differences to the healthy group regarding the delineated area (+1.79 cm^2^, *p* = 0.01) and average speed (+5.17 mm/s, *p* = 0.007) (Table 2).

The correlation coefficients with the EDSS were significant for all three balance parameters. The delineated area showed the strongest correlation according to Spearman with *r* = 0.427 (*p* < 0.001) (Table 3). The parameters delineated area and average speed had a very strong correlation (*r* = 0.817, *p* < 0.001).

**Table 3:** Correlation coefficients between balance parameters and EDSS step scores, cerebellar function system and romberg test in MS patients (*N* = 99) according to spearman.

	**EDSS step score**	**Cerebellar function system**	**Romberg test**
Delineated area	*r* = 0.427 (*p* < 0.001)	*r* = 0.404 (*p* < 0.001)	*r* = 0,537 (*p* < 0.001)
Average sway	*r* = 0.330 (*p* < 0.001)	*r* = 0.131 (*p* = 0.201)	*r* = 0.153 (*p* = 0.131)
Average speed	*r* = 0.334 (*p* < 0.001)	*r* = 0.349 (*p* < 0.001)	*r* = 0.431 (*p* < 0.001)

### Balance Parameters According to Cerebellar Function Score and Romberg Test

**Figure 1:**
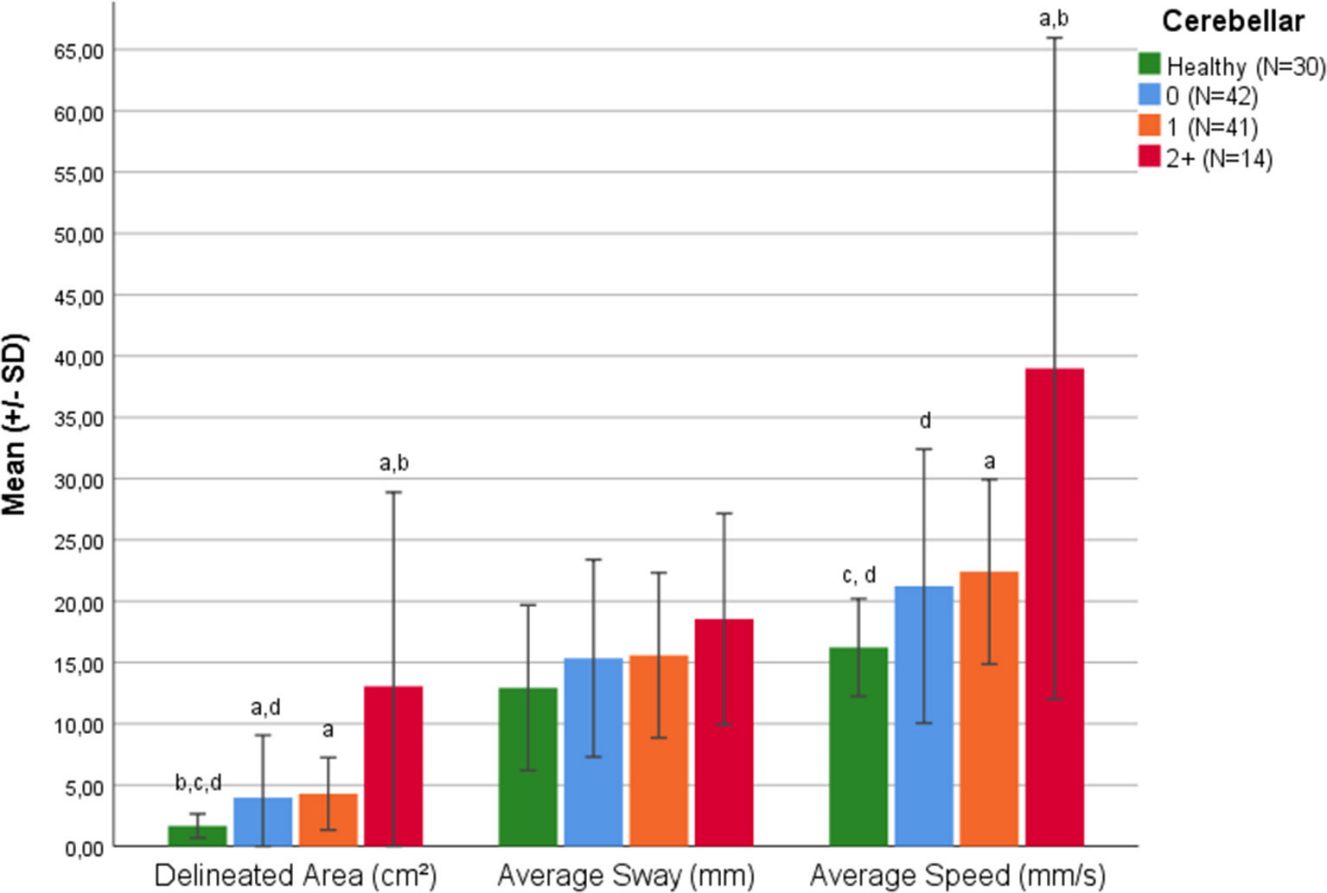
Balance parameters in healthy subjects and in pwMS classified according to Cerebellar Function Score. a = significant difference to healthy group (*p* < 0.05). b = significant difference to Romberg Score 0 (*p* < 0.05). c = significant difference to Romberg Score 1 (*p* < 0.05). d = significant difference to Romberg Score 2+ (*p* < 0.05).

Considering just the cerebellar functional system of the EDSS, the delineated area and the average speed differed between HS and pwMS subgroups *F*_(3,123)_ = 11.16, *p* < 0.001 and *F*_(3,123)_ = 11.97, *p* < 0.001, respectively. Games-Howell *post-hoc* analysis revealed a significant difference between HS and pwMS, with a cerebellar score of 0 in the delineated area (+0.519 cm^2^, *p* = 0.032) (Figure 1). This parameter and the average speed of sway could differentiate pwMS with a cerebellar score of 1 from HS as well (*p* < 0.001) (Figure 1).

**Figure 2:**
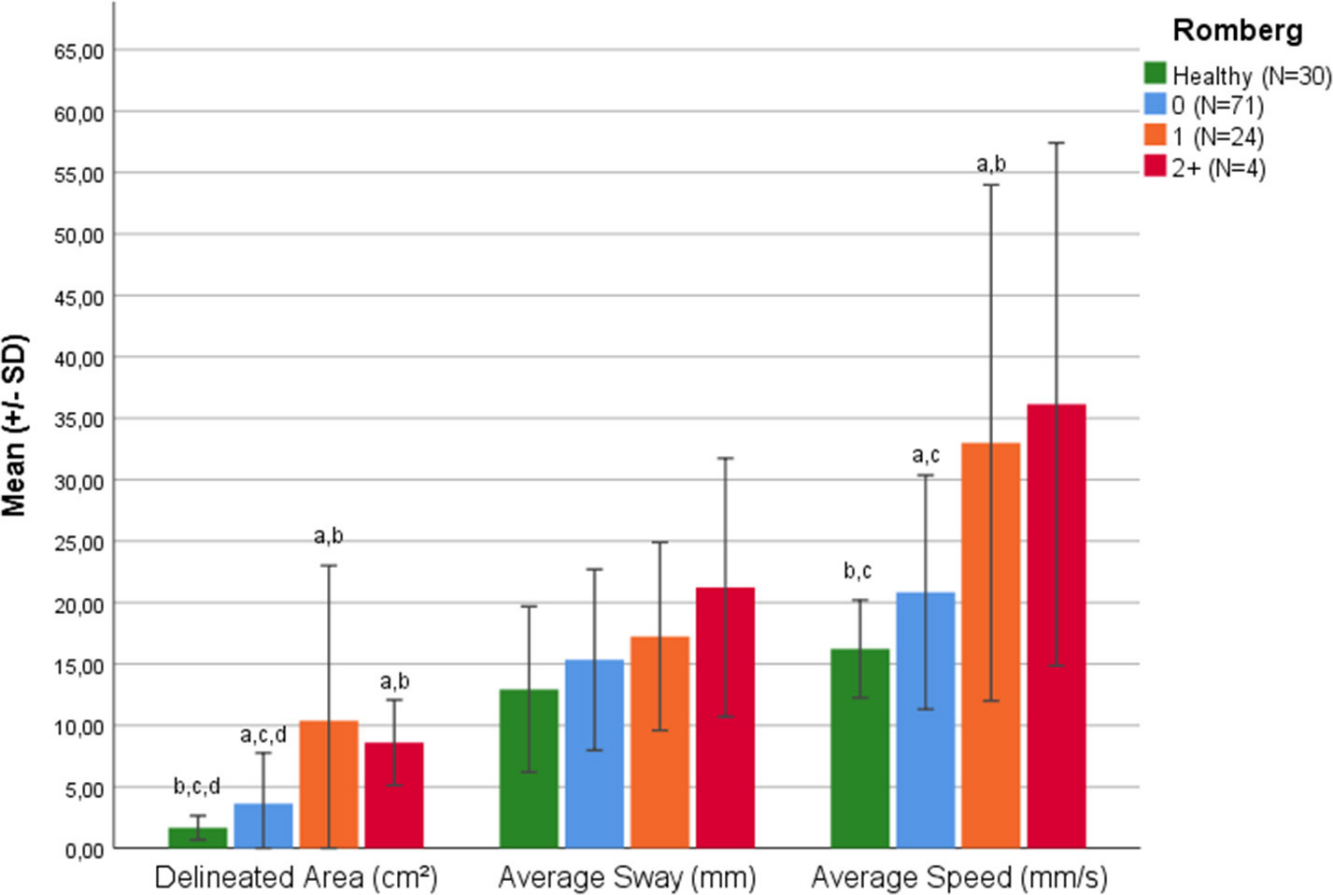
Balance parameters in healthy subjects and pwMS classified according to Romberg Test. a = significant difference to healthy group (*p* < 0.05). b = significant difference to Cerebellar Score 0 (*p* < 0.05). c = significant difference to Cerebellar Score 1 (*p* < 0.05). d = significant difference to Cerebellar Score 2+ (*p* < 0.05).

Similarly, the delineated area and average speed could differentiate pwMS according to Romberg test score and HS, namely *F*_(3,125)_ = 10.08, *p* < 0.001 and *F*_(3,125)_ = 10.86 *p* < 0.001, respectively. Additionally, HS differed from pwMS with a Romberg test score of 0 in delineated area (+0.56 cm^2^, *p* < 0.001) and average speed (+0.200 mm/s, *p* = 0.008) (Figure 2). The mentioned parameter, as well as the average speed could also differentiate HS from MS patients with a Romberg score of 1 (*p* < 0.001).

Both delineated area and average speed were also significantly correlated to the cerebellar function system score and Romberg test (*p* < 0.001) (Table 3).

### Patients With Normal Cerebellar Function and Romberg Tests and Impaired Balance Parameters

An additional analysis was performed to detect the number of pwMS with normal cerebellar function system score and Romberg tests that had impaired balance parameters compared to HS. Three cut-points were evaluated. Considering a strict limit of 3 standard deviations from the healthy group, 21.43% (9 out of 42) of pwMS with a normal cerebellar function score according to the neurologist had an impaired delineated area and 19.05% had an impaired average speed of sway (Table 4).

**Table 4:** Patients with cerebellar function system score 0 (*N* = 42) or Romberg test score 0 (*N* = 71) with impaired balance parameters according to different cut-points for deviation from the healthy group.

		**2 SD**	**2.5 SD**	**3 SD**
Cerebellar function system score = 0 (*N* = 42)	Delineated area	13 (30.95%)	10 (23.81%)	9 (21.43%)
	Average sway	6 (14.29%)	2 (4.76%)	1 (2.38%)
	Average speed	10 (23.81%)	8 (19.05%)	8 (19.05%)
Romberg test score = 0 (*N* = 71)	Delineated area	39 (54.93%)	30 (42.25%)	28 (39.44%)
	Average sway	13 (18.31%)	5 (7.04%)	2 (2.82%)
	Average speed	26 (36.62%)	19 (26.76%)	19 (26.76%)

Similar results can be seen regarding the Romberg test score. Among those with a score of 0, up to 39.44% (28 out of 71) had an impaired delineated area and 26.76% an altered average speed of sway with a limit of 3 SD from the healthy group. A great proportion of patients had still impaired values considering stricter limits (Table 4).

## Discussion

In this study, we were able to analyze different balance parameters in healthy subjects and patients with different disability degrees. Our MS group was typical for MS patients considering age and sex ratio. We characterized our study population with the most used clinical scoring scale in MS (EDSS), including its cerebellar function system score and the Romberg test. With static posturography, balance impairment could be detected even in patients without disability according to the neurological examination.

The first hints of subtle changes in postural stability came from a study by Karst et al., which confirmed the suitability of posturographic processes for long-term observation of standing stability in only slightly impaired MS patients (34).

As expected, as the neurological disability increases, the postural balance is progressively impaired. Patients with higher EDSS scores needed a larger area for standing than healthy subjects or patients with a lower score, confirming previous reports that postulated growing standing instability with an increase in the severity of clinical impairment (4, 23, 26–28).

Our study was also able to demonstrate significant differences in balance parameters between MS patients with minimal EDSS scores (1.0–1.5) and healthy subjects. These results vary slightly from previous reports, where there was no difference in patients with an EDSS score < 2.0 compared to healthy subjects (28). Further, considering just the cerebellar function system and the Romberg test, a difference in the delineated area and average speed of sway between MS patients and healthy subjects was already detected in the cerebellar function system and Romberg tests, even in those with values of 0 or 1 in these tests.

In the scoring of the EDSS, patients assessed with a 0 have no signs of clinical disease detected by the physician; those with a score of 1 may have signs only of the disease but no disability on daily activities. With static posturography, balance alterations could be detected before they were perceivable by either the physician or the patient according to the EDSS and the evaluated cerebellar function and Romberg test.

Even with the strictest cut-point definitions, up to 21.43 and 39.44% of patients had impaired delineated area values, even if a normal cerebellar function respective Romberg test was previously determined by the physician. Similar results were reported by Melillo et al., who detected balance abnormalities in several patients with normal Romberg test scores with possible prediction of balance impairment after a 1-year follow-up (24). Our results are therefore in agreement with previous publications that indicate a better sensibility of balance parameters than trained neurologists (24, 35, 36).

Additionally, the delineated area and the average speed of sway had moderate correlations with the EDSS, cerebellar function system score and Romberg test, confirming preceding findings (26, 27, 36, 37). Previous studies could even predict EDSS scores using static posturography (29).

The correlation between the delineated area and the average sway was high (*r* = 0.817). However, both were less correlated with the average sway (*r* = 0.424 and *r* = 0.457, respectively). This is in line with the results reported in our study, as the average sway had the lowest sensitivity detecting impairment between pwMS and HS. This may be a consequence of specific technical characteristics of the GK-1000 Force Platform available for this study related to the used piezoelectric sensors responsible for the measurement of the patient's center of gravity and its projection on the field. The average sway was a unidimensional outcome measure (mm) and could have a lower sensibility. The delineated area and the average sway could represent a support for physicians assessing disease impairment and addressing further therapeutic procedures, especially in patients with low or undetectable disability.

However, some limitations of our study should be considered. Firstly, different force platforms as well as different techniques for static posturography are currently available, and results could vary according to the methods used for the calculation of balance parameters. We consider the ideal solution for increasing comparability between different studies to be the use of precisely similar force platforms and extraction software. The use of commercially distributed systems could unify outcomes obtained from pwMS. Second, there are no reference values available for our used platform, which can support the detection of imbalance. We proposed the use of standard deviations from a HS group for this goal. Further analysis with more representative HS groups should be performed. However, our HS and pwMS groups showed similar age and gender ratio. Third, we conducted a cross-sectional study. Future research should focus on a longitudinal evaluation which might provide further insights into the utility and prognostic value of the used technique.

The results of our study support the use of static posturography in clinical practice. This technique could be useful to address preventive strategies in patients with low disability and prevent further falls and lesions due to imbalance (26, 38, 39). Ideally, therapeutic interventions should be introduced even before postural stability deficits become clinically relevant, which is precisely why the development of reliable diagnostic procedures for the early detection of walking and standing instability is relevant in clinical practice (34). Future approaches could consider concomitant MS impairments such as cognitive dysfunction and their effect on postural control using static posturography. PwMS could be more unbalanced by adding cognitive tasks and an improvement of balance function after multi-tasking training has been reported (40–42).

All three evaluated parameters were able to differentiate pwMS from HS. Nevertheless, just the delineated area and average speed could detect differences between HS and pwMS with normal cerebellar function and Romberg test. They could therefore be used for future studies and examinations where balance is concerned.

Static posturography parameters could moreover be used as outcome measures in clinical trials, complementing the EDSS with additional advantages regarding psychometric characteristics of its execution. Results are obtained on a continuous linear scale, with better reliability as it is less operator-dependent and possibly associated with greater sensitivity compared to the EDSS.

Overall, the balance platform test seems suitable for assessing the current postural stability of MS patients. Future studies should evaluate the responsiveness or sensitivity to change, in order to determine if it could be used for the development of a standardized measurement for the middle and long-term follow-up of disease progression and for treatment response evaluation toward the digitalization and objective assessment in medicine.

## Conclusion

Balance parameters obtained with static posturography were able to discriminate between MS patients and healthy subjects, even without disability detected by a physician using the EDSS and the Romberg test. Specifically, the delineated area and average speed of sway measured with the patient standing with eyes closed are sensitive parameters for the assessment of balance impairment in early stages of the disease. These tools could complement the EDSS and neurological examination for a more sensitive and objective assessment of MS patients.

## Data Availability Statement

The raw data supporting the conclusions of this article will be made available by the authors, without undue reservation, to any qualified researcher.

## Ethics Statement

The studies involving human participants were reviewed and approved by Ethikkommission an der Technical University Dresden. Approval number: EK 224062011. The patients/participants provided their written informed consent to participate in this study.

## Author Contributions

HI, DS, AK, KT, and TZ contributed conception and design of the study and wrote sections of the manuscript. HI and DS organized the database and performed the statistical analysis. TZ, DS, and HI wrote the first draft of the manuscript. All authors contributed to manuscript revision, read, and approved the submitted version.

### Conflict of Interest

TZ received personal compensation from Biogen, Bayer, Celgene, Novartis, Roche, Sanofi, and Teva for consulting services. TZ received additional financial support for the research activities from Bayer, BAT; Biogen, Novartis, Teva, and Sanofi. The remaining authors declare that the research was conducted in the absence of any commercial or financial relationships that could be construed as a potential conflict of interest.
